# Presynaptic Muscarinic Acetylcholine Receptors and TrkB Receptor Cooperate in the Elimination of Redundant Motor Nerve Terminals during Development

**DOI:** 10.3389/fnagi.2017.00024

**Published:** 2017-02-08

**Authors:** Laura Nadal, Neus Garcia, Erica Hurtado, Anna Simó, Marta Tomàs, Maria A. Lanuza, Victor Cilleros, Josep Tomàs

**Affiliations:** Unitat d’Histologia i Neurobiologia (UHN), Facultat de Medicina i Ciències de la Salut, Universitat Rovira i VirgiliReus, Spain

**Keywords:** motor nerve terminal, cholinergic synapses, neuromuscular junction, presynaptic muscarinic acetylcholine receptors, TrkB receptor, synapse elimination

## Abstract

The development of the nervous system involves the overproduction of synapses but connectivity is refined by Hebbian activity-dependent axonal competition. The newborn skeletal muscle fibers are polyinnervated but, at the end of the competition process, some days later, become innervated by a single axon. We used quantitative confocal imaging of the autofluorescent axons from transgenic B6.Cg-Tg (Thy1-YFP)16 Jrs/J mice to investigate the possible cooperation of the muscarinic autoreceptors (mAChR, M_1_-, M_2_- and M_4_-subtypes) and the tyrosine kinase B (TrkB) receptor in the control of axonal elimination after the mice *Levator auris longus (LAL)* muscle had been exposed to several selective antagonist of the corresponding receptor pathways *in vivo*. Our previous results show that M_1_, M_2_ and TrkB signaling individually increase axonal loss rate around P9. Here we show that although the M_1_ and TrkB receptors cooperate and add their respective individual effects to increase axonal elimination rate even more, the effect of the M_2_ receptor is largely independent of both M_1_ and TrkB receptors. Thus both, cooperative and non-cooperative signaling mechanisms contribute to developmental synapse elimination.

## Introduction

During the development of the nervous system, synapses are eliminated on a broad scale (Thompson, 1985; Bourgeois and Rakic, 1993). This allows connectivity to be refined on the basis of Hebbian activity-dependent axonal competition (Jansen and Fladby, 1990; Sanes and Lichtman, 1999). In newborn animals, the skeletal muscle fibers are polyinnervated in the neuromuscular junction area (NMJ; Redfern, 1970; Brown et al., 1976; Ribchester and Barry, 1994), but at the end of the axonal competition, endplates are innervated by a single axon (Benoit and Changeux, 1975; O’Brien et al., 1978; Jansen and Fladby, 1990; Sanes and Lichtman, 1999). This peripheral synapse has been studied extensively as a model for synapse development (Liu et al., 1994; Nguyen and Lichtman, 1996; Chang and Balice-Gordon, 1997; Sanes and Lichtman, 1999; Lanuza et al., 2001; Santafé et al., 2001; Herrera and Zeng, 2003; Wyatt and Balice-Gordon, 2003; Buffelli et al., 2004; Garcia et al., 2010). Several signaling molecules and presynaptic receptors play a role in the axonal competition, which means that the various nerve endings influence one another (Santafé et al., 2009a; Garcia et al., 2010; Nadal et al., 2016). Postsynaptic-derived trophic substances (Nadal et al., 2016) and the participation of glial cells (Lee et al., 2016; Yang et al., 2016) also make a decisive contribution.

In a previous study, we investigated how individual muscarinic acetylcholine receptor (mAChR) subtypes (M_1_, M_2_ and M_4_), adenosine receptors (AR; A_1_ and A_2A_) and tropomyosin-related tyrosine kinase B (TrkB) receptors are involved in the control of synapse elimination in the mouse NMJ (Nadal et al., 2016). The data show that mAChR, AR and TrkB signaling lessen the initial chance of axonal elimination (around P5–P7) by extending the period of axonal competition but then increase (around P9) axonal loss rate (Nadal et al., 2016). The three receptor sets promote axonal disconnection at the beginning of the second postnatal week largely independently of the postsynaptic nicotinic acetylcholine receptor (nAChR) cluster maturation. In addition, a real cooperation between some of the mAChR and AR subtypes is observed. More specifically, preliminary results show that both AR subtypes (A_1_ and A_2A_) can add their independent effect on axonal loss to the effect of the M_1_ muscarinic receptor, which leads to greater elimination because of the additive effect of the pathways (Nadal et al., 2016).

In this study, we investigate whether the mAChR subtypes and the TrkB receptor also work together, and whether the respective pathway inhibitors have any additive or occlusive effects and, therefore, whether there is any real cooperation between them in synapse elimination at the NMJ. The main result shows that, like the mAChR and AR relations, the effect of M_1_ and TrkB receptors can be added to increase axonal loss rate at P9 but that the effect of M_2_ is largely independent of the TrkB receptors. Thus, cooperative and non-cooperative signaling contribute to synapse elimination, which highlights the importance of axonal competition and loss in the development of neural connectivity.

## Materials and Methods

### Animals

Transgenic B6.Cg-Tg (Thy1-YFP)16 Jrs/J mice were used (The Jackson Laboratory,Bar Harbor, ME, USA). The mice express spectral variants of GFP (yellow-YFP) at high levels in motor and sensory neurons, and axons are brightly fluorescent all the way to the terminals.

Experiments were performed on the *Levator auris longus* (LAL) muscle. Neonatal pups of either sex (9 days) were obtained and the date of birth was designated postnatal day 0 (P0). We minimized the variability in our measurements by carefully monitoring the timing of conception. Also, the weights of the individuals were within 5% of the mean for a given day after conception. The mice were cared for in accordance with the guidelines of the European Community’s Council Directive of 24 November 1986 (86/609/EEC) for the humane treatment of laboratory animals. All experiments on animals have been reviewed and approved by the Animal Research Committee of the Universitat Rovira i Virgili (Reference number: 0233).

### Injection Procedure

The newborn mice were anesthetized with 2% tribromoethanol (0.15 ml/10 g body weight, i.p.). Mice pups received daily subcutaneous injections in the back of the neck beginning on postnatal day 5 of one or two substances (combinations of two selective mAChR antagonists or of one mAChR antagonist plus the TrkB signaling agent TrkB-Fc). Under aseptic conditions, solutions were administered in 50 μl of sterile physiological saline by subcutaneous injection over the LAL external surface as described elsewhere (Lanuza et al., 2001). The animals received four injections from postnatal day 5, and the LAL muscles were studied on day 9. Control injections were given in exactly the same way as experimental injections, using phosphate buffered saline (PBS) alone. No differences were found between the muscles injected or not with PBS, thus indicating that the injection procedure did not in itself induce changes in the overall morphology of the motor endplate and nerve terminals. The solutions were administered at a concentration in accordance with the previously reported biological action of the substance (Santafé et al., 2004, 2015; Garcia et al., 2010).

### Tissue Preparation and Histochemistry

Neonatal pups were given a lethal dose of 2% tribromoethanol. Their heads were removed and fixed in 4% paraformaldehyde for 1.5 h. After washing in PBS, LAL muscles were removed and post-fixed for 45 min. After washing in PBS, Thy1-YFP LAL muscles were incubated in PBS containing a 1/800 dilution of 1 μg/ml tetramethylrhodamine conjugated α-bungarotoxin (α-BTX-TRITC; T1175, Molecular Probes, Eugene, OR, USA) for 1 h at room temperature. Whole muscles were mounted in Mowiol with p-phenylenediamide (Sigma).

### Confocal Microscopy and Morphological Analysis

NMJs were analyzed using an inverted Nikon TE-2000 fluorescent microscope (Nikon, Tokyo, Japan) connected to a standard personal computer that was running image analysis software (ACT-1, Nikon). The number of axons per endplate was counted. Because of the difficulty of determining the exact number of axonal inputs for each nAChR cluster, when more than two axons converged at the same synaptic site we classified the NMJs into three groups only: junctions that were monoinnervated, doubly innervated, or innervated by three or more terminal axons. These data enabled us to calculate the “average number of axonal inputs” and the “percentage of polyneuronal innervation” for all fibers receiving two or more axons.

### Statistical Analysis

All NMJs visible in their entirety were scored, with a minimum of 100 per muscle. At least six muscles were studied for each age and condition examined. Fisher’s test was applied to compare percentages. The criterion for statistical significance was *P* < 0.05. The categories were scored and the counting was performed by a person with no knowledge of the age or treatment of the animals. The data are presented as mean ± SD.

### Drugs

#### Selective M_1_, M_2_ and M_4_ mAChR Antagonists

The stock solutions were pirenzepine (PIR) dihydrochloride (1071, Tocris Bioscience) 10 mM; methoctramine (MET; M105, Sigma—Aldrich, St. Louis, MO, USA) 1 mM; muscarinic toxin 3 (MT3; M-140, Alomone Labs) 50 μM. The working solutions used were PIR (10 μM), MET (1 μM) and MT3 (100 nM).

#### TrkB Receptor-Related Agent

The following stock solutions were used: recombinant human trkB/Fc Chimera (trkB-Fc; 688-TK; R&D Systems), 100 μg/ml. Working solutions were trkB-Fc 5 μg/ml.

## Results

### mAChR and TrkB Receptors in Axon Loss Control

**Figure 1:**
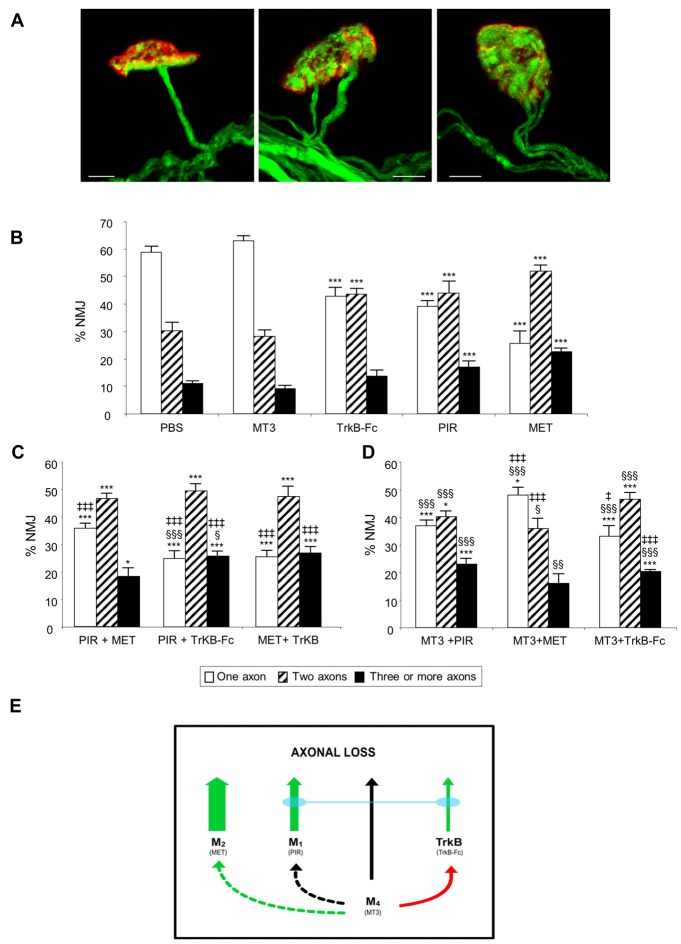
**The picture in (A)** shows representative confocal immunofluorescence images of singly-, dually- and polyinnervated neuromuscular junction area (NMJ) from P9 YFP mice. Scale bar: 10 μm. The histograms in **(B)** show the percentage of singly-, doubly- and triply- (or more) innervated NMJs in control (phosphate buffered saline, PBS treated) and levator auris longus (LAL) muscles treated with the inhibitors considered. We show newly reproduced data of previous results to facilitate comparison (Nadal et al., 2016). **(C)** shows the percentage of NMJs after the simultaneous inhibition of two receptors clearly involved in axonal elimination (those that affect axon loss rate when they are individually blocked (all except M_4_). The associations of the M_4_ blocker muscarinic toxin 3 (MT3) with the other inhibitors are represented in **(D)**. An overall representation of the data illustrating the individual role and cooperation of the muscarinic acetylcholine receptor (mAChR) and tyrosine kinase B (TrkB) receptors in developmental axonal loss modulation is shown in the diagram in **(E)**. The green arrows show how effective these receptors are at accelerating axonal elimination (the thicker they are, the greater their effect). The association of the M_1_ and TrkB blockers results in the addition of their respective effects (blue bond between these receptors). The M_4_ receptor, which does not by itself affect axonal elimination (black arrow), cooperates positively with M_2_ (dotted green arrow) and produces some occlusion of the TrkB pathway (red arrow) but does not cooperate with M_1_ (dotted black arrow). All NMJs visible in their entirety were scored, with a minimum of 100 synapses per muscle. At least six muscles were studied for each age and condition examined. Fisher’s test was applied to compare percentages. When the corresponding antagonist or combinations of two substances were compared with control PBS, significance symbols are: *P < 0.05, ***P < 0.005. ^§^*P* < 0.05, ^§§^*P* < 0.01, ^§§§^*P* < 0.005 when the combination of two substances were compared with the first. ^‡^*P* < 0.05, ^‡‡‡^*P* < 0.005 when the combination of two substances were compared with the second.

Figure 1A shows representative confocal immunofluorescence images of the autofluorescent axons in singly- and polyinnervated LAL P9 NMJs from B6.Cg-Tg (Thy1-YFP) mice (hereafter YFP). When the mAChR subtype-selective inhibitors PIR (M_1_ blocker) and MET (M_2_ blocker), and the TrkB pathway blocker (a TrkB-Fc chimera) were applied once a day between P5-P8 on the LAL muscle surface, there was a notable delay in the transition to monoinnervation on the NMJ observed at P9 (Nadal et al., 2016). However, the M_4_ subtype blocker MT3 shows no effect on axonal loss at this time. Figure 1B shows the effect of these selective blockers in increasing order of their relative ability to delay monoinnervation and maintain a high percentage of synapses innervated by three or more axons (PBS-P9 = MT3 < TrkB-Fc < PIR < MET). MET and PIR delayed the three-to-one axon transition whereas TrkB-Fc delayed the two-to-one transition. Therefore, in normal conditions without inhibitors, the two receptor sets (mAChR and TrkB) will contribute to promoting axonal disconnection at the beginning of the second postnatal week (see also Nadal et al., 2016). However, the absolute potency of these receptors in modulating synapse loss cannot be directly compared because the blocking efficacy of the respective selective inhibitors is not the same.

### Cooperation Between mAChR and TrkB Receptors

Our experiments were designed to investigate the effect on axonal loss of simultaneous incubation with two inhibitors (two antagonists of two different receptors) and reveal the possible occlusive or additive crosstalk effects between the corresponding pathways. Figures 1C,D show the effect of the association of the drugs applied between P5 and P8 and observed at P9. Figure 1C shows the percentage of NMJs—of singly-, doubly- and triply- (or more) innervated endplates—after the simultaneous inhibition of two receptors that, individually, clearly modulate axon loss (see Figure 1B; all except M_4_). The associations of the M_4_ blocker MT3 (which does not affect axonal elimination by itself) with the other substances has been represented separately in Figure 1D so that the data is more understandable. The M_4_ subtype is shown to have a complementary role.

The association of the mAChR blockers PIR and MET shows no additive effect or mutual occlusion in relation to axonal loss (Figure 1C). However, the association of the M_1_ and TrkB pathway inhibitors (PIR plus TrkB-Fc) results in a clear addition of their respective delaying effects on axonal loss. The percentage of the monoinnervated NMJ after simultaneous exposure to both inhibitors is significantly less (25% of single junctions) than after exposure to only PIR (39%) or only TrkB-Fc (43%). Interestingly, however, the individual effect of the TrkB-Fc does not add to the effect of MET and the result of this dual drug incubation is no different from the effect of MET by itself. The delaying effect of MET on axon loss is the most potent observed in the present experiments and is produced independently of the state of TrkB.

Figure 1B shows that the M_4_ blocker MT3 by itself has no effect on axonal loss at P9. Figure 1D shows that if MT3 is simultaneously applied with the other blockers it reveal some regulatory or complementary role of M_4_ on the other receptors. The presence of MT3 does not change the effect of PIR although some occlusion of the potent effect of MET is observed. MET still continues to significantly delay axon loss (the three-to-one transition), however. Interestingly, the presence of MT3 potentiates the delaying effect of TrkB-Fc on axonal loss, which indicates that the respective receptor pathways (M_4_ and TrkB) are cooperating.

These data are represented in Figure 1E. The green arrows of different thicknesses show how effective these receptors are at accelerating axonal elimination (the thicker they are, the greater their effect; the inhibitor used is in brackets under the name of the receptor). The M_4_ receptor by itself does not affect axonal elimination at P9 (black arrow). Interestingly, the association of the M_1_ and TrkB pathway blockers results in the addition of their respective delaying effects on axonal loss, which indicates that the corresponding receptors are cooperating (blue bond between these receptor pathways). However, the potent effect of the M_2_ cannot be modified with the simultaneous presence of the M_1_ or TrkB blockers. It seems that the M_4_ receptor, which does not by itself affect axonal elimination, cooperates positively with M_2_ (dotted green arrow). Also, M_4_ produces some occlusion of the TrkB pathway (red arrow) but does not cooperate with M_1_ (dotted black arrow).

## Discussion

The present experiments show evidence of the cooperation between the presynaptic M_1_, M_2_ and M_4_ mAChR subtypes and the TrkB signaling to modulate the conditions of the developmental axonal competition and loss. In a previous study we found that these receptors (as well as presynaptic AR, A_1_ and A_2A_ subtypes) separately contribute to accelerate synapse elimination around P9 in the mouse NMJ (Nadal et al., 2016). It was thought that the muscarinic autoreceptors of the transmitter acetylcholine (ACh) may allow direct competitive interaction between nerve endings through a differential activity-dependent ACh release. The more active axons may directly punish the less active ones or reward themselves (Santafé et al., 2009a). However, an axon that is eliminated at one NMJ may be successful at another (Tomàs et al., 2011), which suggests that other receptors and local postsynaptic- (and glial cell) derived factors are involved. The involvement of the TrkB signaling described may allow a postsynaptic-derived trophic substance such as Brain-derived neurotrophic factor (BDNF) or neurotrophin-4 (NT-4) to make a contribution (Yoshii and Constantine-Paton, 2010).

Interestingly, we observed that both the presynaptic-derived signal (ACh acting on axonal M_1_ and M_2_ mAChRs) and the TrkB-mediated signal (which may be originated by a postsynaptic-derived NT) have the same effect: namely, the acceleration of supernumerary nerve ending elimination. It seems that the outstanding regulatory resources in the NMJ synaptogenesis are committed to achieving monoinnervation. These presynaptic receptors converge in a common intracellular mechanism and a limited repertoire of effector kinases to phosphorylate protein targets and bring about structural and functional changes leading to axon loss. It is well known that in most cells M_1_ and TrkB operate by stimulating the phospholipase C gamma (PLC gamma) and therefore the protein kinase C (PKC) pathway along with the inositol triphosphate (IP3) pathway, whereas M_2_–M_4_ inhibit the adenyl cyclase (AC) and protein kinase A (PKA) pathway (Caulfield, 1993; Felder, 1995; Caulfield and Birdsall, 1998; Nathanson, 2000). In all cases, however, common final changes such as intracellular calcium oscillations can occur (Santafé et al., 2006; Amaral and Pozzo-Miller, 2012). Both PKA and PKC activity changes have been shown to affect pre- and postsynaptic maturation (Lanuza et al., 2001, 2002). Our present data can be related with the intracellular coupling of the receptors to these serine kinases. Though the blocking efficacy of the selective inhibitors of the muscarinic receptors is not assessed here, M_2_ increases the axonal loss rate most with a slight involvement of the M_4_ receptor but independently of the M_1_ and TrkB receptors. This suggests that downregulation of PKA activity through the couple M_2_–M_4_ is a key factor in synapse elimination. Concurrently, M_1_ and TrkB also contribute separately to axonal loss, but their combined action has a potent summed effect similar to the effect of the M_2_ receptor. This suggest that activation of the PLC gamma-PKC pathway through the couple M_1_-TrkB may be the other key factor in this process. Thus, a displacement of the PKA/PKC activity ratio to lower values (inhibition of PKA and/or stimulation of PKC) in some nerve endings may have a leading role in synapse elimination. In this context, blockade of PKC in the newborn LAL muscle produces an initial blockade of synapse elimination and a subsequent delay (Lanuza et al., 2002).

In fact, these changes in the kinase activity leading to synapse elimination must occur at least (but not only) in the weakest axons during the competitive interactions. The neurotransmitter release capacity is an important factor in the competing capacity of the various nerve terminals in a NMJ. During development, in the polyinnervated NMJ several nerve endings with different levels of maturation and ACh release capacity get together and compete. The coupling to neurotransmitter release of the considered receptors and kinases is not the same in each of these various endings themselves and in the mature synapses in the adult (Tomàs et al., 2014). So, how does the specific coupling to ACh release of receptors and kinases in the weak and strong axons in competition contribute to axonal loss? As far as serine kinases are concerned, in the adult motor nerve endings both PKA and PKC potentiate ACh release when coupled to neurotransmission (Santafé et al., 2009b). Similarly, the same potentiation is observed in most neuromuscular synapses during development as, for instance, in those formed by the strongest axons (those that evoke the large endplate potential, EPP) in the polyinnervated junctions (Santafé et al., 2004). However, in the weakest endings the inhibition of PKC increases the evoked EPP size indicating that, in normal conditions without any inhibition, this kinase tonically couples to ACh release reduction in these low releasing synapses. Therefore, an M_1_-TrkB-mediated increase in PKC activity in the weakest endings would debilitate further their ACh release capacity and competitive force and facilitate their elimination. In addition, an M_2_-mediated PKA downregulation in all nerve endings in competition may differentially affect their ACh release and contribute to elimination. Thus, at this point, there is a significant agreement between the known involvement of these molecules in neurotransmission and axon loss.

However, when considering the real postnatal coupling to ACh release of the mAChR and the TrkB receptor in the different nerve endings (the strongest and the weakest) on developing synapses (Santafé et al., 2004; Garcia et al., 2010), additional interpretative keys are needed. In the mature NMJ, M_1_ and M_2_ subtypes modulate evoked transmitter release by positive and negative feedbacks, respectively (Santafé et al., 2003, 2006). However, during NMJ synaptogenesis, the functional significance of the subtypes is different from in the adult. M_2_ receptors promote release in all nerve endings independently of their ACh release level or maturation state whereas an M_1_- and M_4_-mediated reduction in release is observed in the weakest endings on dual junctions (Santafé et al., 2001, 2002, 2003, 2004, 2007, 2009a). Similarly, the BDNF-TrkB pathway contributes to potentiate ACh release in different neuromuscular adult models but the potentiation is not observed in the weakest nerve endings during development and even some ACh release inhibition was observed in the strongest endings (Garcia et al., 2010). Therefore, interpreting the links and molecular relations between transmitter release and elimination of nerve terminals seems more complex than it seemed at first. The involvement of other signaling such as AR can contribute to this complexity (Todd and Robitaille, 2006; Nadal et al., 2016). However, some conclusions can be drawn on the basis of all the above data. First, it should be pointed out that, contrary to what happens in the adult, M_1_ (and M_4_) and PKC activity reduces ACh release in the weakest endings and promotes axonal loss. In fact, blocking mAChRs (M_1_- and/or M_4_-subtypes) or PKC or voltage-dependent calcium channels (VDCCs; P/Q-, N- or L-type or Ca^2+^ influx) can lead to similar percentage increases in the size of the synaptic potentials evoked by weak axons (Santafé et al., 2003, 2004, 2007, 2009a,b; Tomàs et al., 2011). Therefore, the M_1_-PKC pathway may debilitate the ACh release capacity and competitive force of these synaptic contacts and facilitate their elimination. The final target molecules involved may be the VDCC, specially the L-type which is exclusively coupled to ACh release in these weak endings (Santafé et al., 2001) and may contribute to carry high calcium near the molecular mechanism directly involved in axon loss. Second, like M_1_ signaling, BDNF-TrkB signaling accelerates axon loss. However, it is not so clear whether it is involved in the modulation of ACh release in the nerve endings that are in competition because it does not affect release in the weak axons. Because PKC effectively reduces release in these endings, the TrkB pathway may operate through the IP3 pathway to increase intracellular calcium and modulate the loss of axons. Third, M_2_ promotes axonal elimination the most. However, unlike the adult, this muscarinic subtype promotes ACh release in all the endings that are in competition, including the weakest endings and the solitary ending that finally wins the competition. Therefore, there is a shift in the M_2_ coupling during development but how this affects its relation with PKA and how this relates with axonal loss is not known.

## Author Contributions

LN: data collection, quantitative analysis, literature search and data interpretation; EH, AS and MT: data collection, NG: statistics; JT, NG and MAL: conception and design, literature search, data interpretation, confocal microscopy and manuscript preparation. JT, NG and MAL contributed equally to this work. All authors listed, have made substantial, direct and intellectual contribution to the work, and approved it for publication.

## Funding

This work was supported by a grant from the Catalan Government (2014SGR344) and a grant from Ministerio de Economía y Competitividad (MINECO; SAF2015-67143-P).

## Conflict of Interest Statement

The authors declare that the research was conducted in the absence of any commercial or financial relationships that could be construed as a potential conflict of interest.

## Abbreviations

AC: adenyl cyclase
ACh: acetylcholine
AR: adenosine receptors
BDNF: Brain-derived neurotrophic factor
EPP: evoke endplate potentials
IP3: inositol triphosphate
LAL: Levator auris longus muscle
mAChR: muscarinic acetylcholine receptor
M_1_: M_1_-type muscarinic acetylcholine receptor
M_2_: M_2_-type muscarinic acetylcholine receptor
M_4_: M_4_-type muscarinic acetylcholine receptor
MET: methoctramine
MT-3: muscarinic toxin 3
nAChR: nicotinic acetylcholine receptor
NMJ: neuromuscular junction
NT-4: neurotrophin-4
PBS: phosphate buffered saline
PIR: pirenzepine
PKA: protein kinase A
PKC: protein kinase C
PLC: phospholipase C
TrkB: tropomyosin-related kinase B receptor
VDCC: voltage-dependent calcium channels.
